# Systematic evaluation of IgG responses to SARS-CoV-2 spike protein-derived peptides for monitoring COVID-19 patients

**DOI:** 10.1038/s41423-020-00612-5

**Published:** 2021-01-22

**Authors:** Yang Li, Dan-yun Lai, Qing Lei, Zhao-wei Xu, Feng Wang, Hongyan Hou, Lingyun Chen, Jiaoxiang Wu, Yan Ren, Ming-liang Ma, Bo Zhang, Hong Chen, Caizheng Yu, Jun-biao Xue, Yun-xiao Zheng, Xue-ning Wang, He-wei Jiang, Hai-nan Zhang, Huan Qi, Shu-juan Guo, Yandi Zhang, Xiaosong Lin, Zongjie Yao, Pengfei Pang, Dawei Shi, Wei Wang, Xiao Yang, Jie Zhou, Huiming Sheng, Ziyong Sun, Hong Shan, Xionglin Fan, Sheng-ce Tao

**Affiliations:** 1 Shanghai Center for Systems Biomedicine, Key Laboratory of Systems Biomedicine (Ministry of Education), Shanghai Jiao Tong University, Shanghai, China; 2 Department of Pathogen Biology, School of Basic Medicine, Tongji Medical College, Huazhong University of Science and Technology, Wuhan, China; 3 Key Laboratory of Gastrointestinal Cancer (Fujian Medical University), Ministry of Education, School of Basic Medical Sciences, Fujian Medical University, Fuzhou, China; 4 Department of Clinical Laboratory, Tongji Hospital, Tongji Medical College, Huazhong University of Science and Technology, Wuhan, China; 5 BGI Education Center, University of Chinese Academy of Sciences, Shenzhen, 518083 China; 6 Tongren Hospital, Shanghai Jiao Tong University School of Medicine, Shanghai, China; 7 BGI-Shenzhen, Shenzhen, 518083 China; 8 Department of Public Health, Tongji Hospital, Tongji Medical College, Huazhong University of Science and Technology, Wuhan, China; 9 Center for Interventional Medicine, The Fifth Affiliated Hospital of Sun Yat-sen University, Zhuhai, 519000 China; 10 National Institutes for Food and Drug Control, Beijing, 100050 China; 11 Foshan Fourth People’s Hospital, Foshan, 528000 China; 12 Key Laboratory of RNA Biology, Institute of Biophysics, Chinese Academy of Sciences, Beijing, 100101 China

**Keywords:** SARS-CoV-2, Spike, peptide, Antibody, Diagnosis, Diagnostic markers, Adaptive immunity

## Abstract

Serological tests play an essential role in monitoring and combating the COVID-19 pandemic. Recombinant spike protein (S protein), especially the S1 protein, is one of the major reagents used for serological tests. However, the high cost of S protein production and possible cross-reactivity with other human coronaviruses pose unavoidable challenges. By taking advantage of a peptide microarray with full spike protein coverage, we analyzed 2,434 sera from 858 COVID-19 patients, 63 asymptomatic patients and 610 controls collected from multiple clinical centers. Based on the results, we identified several S protein-derived 12-mer peptides that have high diagnostic performance. In particular, for monitoring the IgG response, one peptide (aa 1148–1159 or S2–78) exhibited a sensitivity (95.5%, 95% CI 93.7–96.9%) and specificity (96.7%, 95% CI 94.8–98.0%) comparable to those of the S1 protein for the detection of both symptomatic and asymptomatic COVID-19 cases. Furthermore, the diagnostic performance of the S2–78 (aa 1148–1159) IgG was successfully validated by ELISA in an independent sample cohort. A panel of four peptides, S1–93 (aa 553–564), S1–97 (aa 577–588), S1–101 (aa 601–612) and S1–105 (aa 625–636), that likely will avoid potential cross-reactivity with sera from patients infected by other coronaviruses was constructed. The peptides identified in this study may be applied independently or in combination with the S1 protein for accurate, affordable, and accessible COVID-19 diagnosis.

## Introduction

COVID-19, which is caused by SARS-CoV-2,^1,2^ is a global pandemic. By November 28, 2020, 61,592,095 cases has been diagnosed, with 1,441,936 deaths (https://coronavirus.jhu.edu/map.html).^3^ To bring the pandemic under control, one of the essential tasks is to perform fast, reliable, and affordable diagnosis. Although the nucleic acid test (NAT) is the reference standard for diagnosing COVID-19 with high sensitivity and accuracy, false negative results are commonly observed.^4,5^ Immunological/serological tests for monitoring SARS-CoV-2-specific IgG and IgM responses provide important information to improve the accuracy of diagnosis.^4,5^ In addition, serological tests are suitable for population screening in high-risk regions or among close contacts of patients as well as for surveillance of pandemic spread and assessment of the infection rate in the general population.^6–8^ Moreover, the antibody response has been reported to be associated with disease severity and clinical outcomes.^9,10^

The S protein is the preferential antigen for serological assays. The key reagent of the S protein-based serological assay is recombinant S protein. However, S protein production is difficult and costly^11^; in addition, inconsistencies among different manufacturers or even between batches might contribute to variability in commercial assays using the same antigen.^7,12^ The limited production capacity and high cost of recombinant protein preparation are bottlenecks, particularly for remote regions or impoverished countries. Another consideration is that cross-reactivity due to infection by other human coronaviruses may cause false positive results, especially for four common cold-causing coronaviruses, HCoV-OC43, HKU1, NL63, and 229E, which circulate in the population.^4,11,13^ It has also been reported that S1 exhibits less cross-reactivity than the full-length S protein due to the lower similarity of the S1 subunit among human coronaviruses compared to that of S2.^4^ To develop highly specific serological tests, greater efforts are needed to identify the sections of the S protein that are highly immunogenic and less homologous to those of related coronaviruses.^6,11^

Spike protein-derived peptides that can elicit antibodies in COVID-19 patients have been reported in several studies,^14–18^ including one of our previous works on epitope mapping in a small sample cohort.^19^ For instance, high positivity rates for antibodies against S2–78 (aa 1148–1159) and S2–22 (aa 812–823) are detected in COVID-19 patients. Nevertheless, whether these peptides are suitable for use in diagnosis is still unknown. To fully evaluate the diagnostic value of S protein-derived peptides, this study examined sera from four cohorts, consisting of 2434 serum samples from 858 COVID-19 patients, 63 asymptomatic patients, and 610 controls. Eight peptides were verified to have high potential for use in diagnosis, especially peptide S2–78 (aa 1148–1159), with a diagnostic performance comparable to that of the S1 protein in COVID-19 patients and individuals with asymptomatic infection. By combining the four SARS-CoV-2-specific peptides, we constructed a panel to potentially achieve more specific detection of infection by this coronavirus.

## Results

### Four independent cohorts of samples were collected and designed

To fully evaluate the diagnostic potential of spike-derived peptides, sera from four cohorts of COVID-19 patients and controls from multiple medical centers in China were collected (Table 1). (1) Cohort 1 consisted of 55 sera from convalescent COVID-19 patients and 18 controls.^19^ (2) Cohort 2 included 2360 sera from 784 in-hospital COVID-19 patients and 542 sera from a variety of controls. To accurately evaluate peptides for diagnosis, one serum sample for each patient that was collected at least 21 days after the onset of symptoms was selected according to the suggestion of the WHO (World Health Organization) for antibody laboratory tests.^20^ As a result, 729 sera were selected. The control groups included two types of samples. The first type included sera collected from hospitals and consisted of samples from healthy people (*n* = 92) and patients with upper respiratory infections (URI, *n* = 104), autoimmune diseases (AID, *n* = 120), lung cancer (*n* = 41) and other diseases (*n* = 112), including cardiovascular or cerebrovascular diseases (34.2%), diabetes (9%), nonlung cancers (7.2%) and other conditions. The group of autoimmune disease patients was composed of 72 systemic lupus erythematosus (SLE), 7 rheumatoid arthritis (RA), 9 dermatomyositis (DM), 11 Behcet’s disease (BD), 12 ankylosing spondylitis, and 9 Sjogren syndrome patients. The second type was negative reference sera provided by the National Institutes for Food and Drug Control, including N11-N25 of the National Reference Panel for 2019-nCoV (SARS-CoV-2) IgG Antibody Detection Kit (370096-202001) and other identified controls. (3) Cohort 3 included 19 COVID-19 patients from another hospital and 50 healthy controls. (4) Cohort 4 consisted of asymptomatic cases defined as positive either by the NAT (nucleic acid test) or an antibody test using a commercial assay and a SARS-CoV-2 exposure history (see “Methods”).

**Table 1:** Patients and controls involved in this study

	Cohort 1		Cohort 2			Cohort 3		Cohort 4
Group	COVID-19	Control	COVID-19	Control-1	Control-2	COVID-19	Control	Asymptomatic patient^a^
Numbers	55	18	729	469	73	19	50	63
Stage	Convalescent	—	In hospital	—	—	Convalescent	—	—
Age	41.5 ± 14.9	50.4 ± 12.5	61.4 ± 14.5	53.7 ± 20.6	N/A	53.9 ± 13.2	46.7 ± 20.2	44.9 ± 16.5
Sex								
Male	27	8	361	224	N/A	10	24	25
Female	28	10	368	245	N/A	9	26	38
Severity								
Severe	0	—	393	—	—	0	—	—
Nonsevere	55		336			19		
Outcome								
Discharge	55	—	679	—	—	19	—	—
Death	0		50			0		
Days after onset	27.5 ± 7.7	—	31.1 ± 8.8	—	—	42.6 ± 9.5	—	—
Source	Foshan 4th Hospital, Guangdong	Ruijin Hospital, Shanghai	Tongji Hospital, Wuhan	Tongren Hospital, Shanghai Ruijin Hospital, Shanghai	National Institutes for Food and DrugControl, Beijing	The Fifth Affiliated Hospital Sun Yat-Sen University, Guangdong	Tongren Hospital, Shanghai	Tongji Hospital, Wuhan
Subtypes and numbers	—	Healthy: 8; LC: 8	This is a subgroup of a cohort of 783 cases with a total of 2,360 longitudinal sera.	Healthy: 92; URI: 104; AID: 120; LC: 41; Other diseases: 112	Negative reference samples	—	—	NAT + IgG-IgM-: 4; NAT + IgG+IgM-: 8; NAT-IgG+IgM+: 23; NAT-IgG+IgM-: 28

*URI* upper-respiratory infection, *AID* autoimmune disease, *LC* lung cancer

^a^Asymptotmatic patients: positive according to the NAT (nucleic acid test) or antibody test (IgG or IgM) performed in the hospital

### Peptide S2–78 (aa 1148–1159) and several other peptides exhibited high diagnostic value

**Fig. 1:**
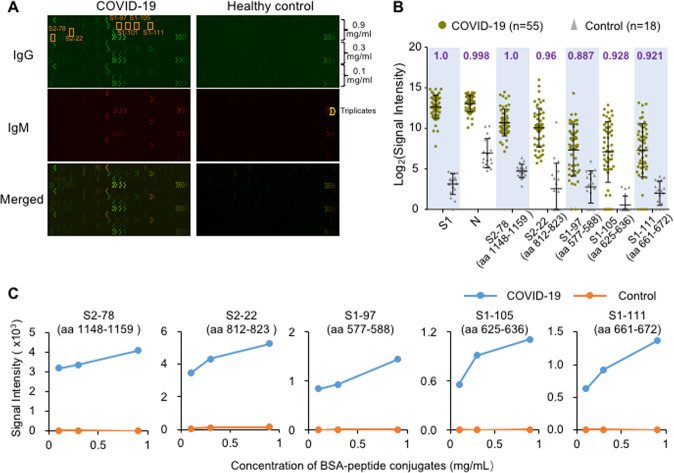
Peptides with strong IgG antibody responses were identified using Cohort 1. **A** Representative images of peptide microarray profiling of sera from a COVID-19 patient and a healthy control. IgG (green) and IgM (red) were detected simultaneously. **B** Box plot of IgG antibody responses to the S1 protein, N protein and other significant peptides for the COVID-19 patient group (*n* = 55) and control group (*n* = 18) of Cohort 1. Each spot indicates one serum sample. Data are presented as box plots, where the middle line is the mean value, and the upper and lower hinges are the mean values ± SD. AUC (area under the curve) values are labeled for each peptide or protein on the top of the box plots. **C** Averaged signal intensities of IgG antibody responses to the indicated peptides at different concentrations, i.e., 0.1, 0.3, and 0.9 mg/mL, for both COVID-19 patients (blue line) and the control group (orange line)

To identify which section of the S protein is of diagnostic importance, it is necessary to survey the entire protein in a systematic way. We previously constructed a peptide microarray with full coverage of the S protein and analyzed 55 convalescent sera from COVID-19 patients along with 18 control sera^19^ (Cohort 1, Table 1). Overall, significant binding of both IgG and IgM was observed in the patient group sera, while the signal was low for the control group sera (Fig. 1A). To statistically and quantitatively analyze the discriminability of the peptides, we calculated the area under the curve (AUC), and the five peptides with the highest AUC values are presented in Fig. 1B. To assess whether the peptide-specific IgG antibody responses are concentration dependent, threefold serially diluted peptides (0.9, 0.3, and 0.1 mg/mL) were printed and immobilized on a microarray. As expected, the average signals of the patient group but not the control group were proportional to the concentrations of the peptides (Fig. 1C).

**Fig. 2:**
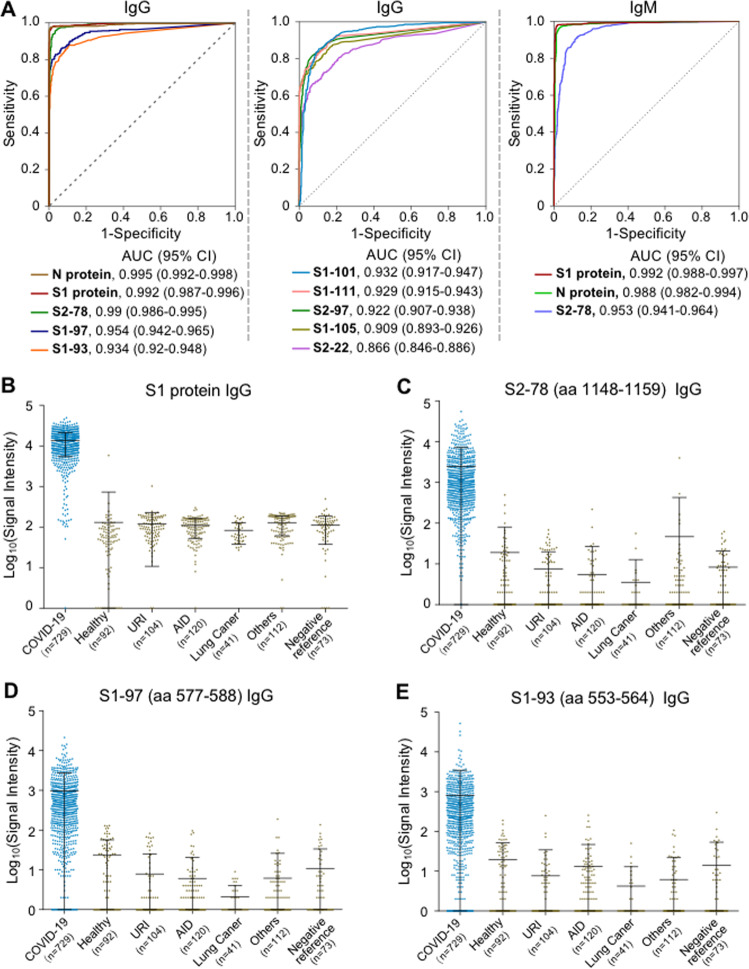
Evaluation of significant peptides using Cohort 2. **A** ROC (receiver operating characteristic) curves of peptides or proteins for discriminating the COVID-19 group (*n* = 729) and the control group (*n* = 542). AUC values with 95% CIs (confidential intervals) are provided for all the peptides and proteins. Signal distributions of anti-S1 IgG (**B**), anti-S2–78 (aa 1148–1159) IgG (**C**), anti-S1–97 (aa 577–588) IgG (**D**) and anti-S1–93 (aa 553–564) (**E**) in the COVID-19 patient (blue) and control groups (yellow). The sample size is indicated for each group. URI upper respiratory infection, AID patients with autoimmune diseases

To extensively evaluate the peptides for diagnostic application, a larger cohort (Cohort 2, Table 1) of samples was screened with a revised peptide microarray that contained only one peptide concentration (0.3 mg/mL) for high-throughput analysis. To ensure that the data generated from the different microarrays were comparable, we prepared a positive reference sample by pooling 50 randomly selected patient sera. This reference sample was then tested using all the microarrays to ensure normalization (see “Methods”). Consistent results were achieved for most of the peptides. The AUC values of IgG or IgM for each peptide were calculated, and eight peptides, S2–78 (aa 1148–1159), S1–97 (aa 577–588), S1–93 (aa 553–564), S1–101 (aa 601–612), S1–111 (aa 661–672), S2–97 (aa 1262–1273), S1–105 (aa 625–636) and S2–22 (aa 812–823), showed high performance; the AUCs of IgG or IgM against these peptides were above 0.85 for both Cohorts 1 and 2. Specifically, for Cohort 2, AUC values with 95% CIs (confidential intervals) for S2–78, S1–97, S1–93, S1–101, S1–111, S2–97, S1–105 and S2–22 were 0.99 (0.986–0.995), 0.954 (0.942–0.965), 0.934 (0.92–0.948), 0.932 (0.917–0.947), 0.929 (0.915–0.943), 0.922 (0.907–0.938), 0.909 (0.893–0.926), and 0.866 (0.846–0.886), respectively (Fig. 2A). For IgM, only S2–78 had an AUC > 0.85 of 0.953 (0.941–0.964) (Fig. 2A). As expected, IgG and IgM against both the N protein and S1 protein exhibited high performance. We further examined antibody responses in different groups for each peptide (Fig. 2B–E, Supplementary Fig. S1). Consistently, the signals for the COVID-19 group were significantly higher than those for the negative control samples in all groups. It was noted that in the group of COVID-19 patients, the signal intensity for S1 IgG was generally higher than that for any single peptide, which may be because there are multiple antibody binding sites on the S1 protein. However, a slightly higher signal was also observed in the control group for protein antigens, demonstrating the occurrence of nonspecific binding; nevertheless, this largely did not occur for the synthetic peptides. Overall, more sensitive detection platforms or higher antigen concentrations might improve the performance of peptides for diagnosis.

### The diagnostic performance of S2–78 (aa 1148–1159) IgG is comparable to that of S1 IgG for COVID-19 patients

We next focused on S2–78 (aa 1148–1159), the peptide with the best detection performance. The optimal Youden index of the ROC (receiver operating characteristic) curve was used to set the cutoff value. The specificity, sensitivity and overall accuracy (95% CI) of S2–78 IgG for the detection of COVID-19 were 96.7 (94.8–98.0%), 95.5% (93.7–96.9%) and 96% (94.8–97%), respectively, slightly lower than those of the S1 IgG (Table 2). Since serological testing is essential for population screening, we calculated the PPV (positive predictive value) and NPV (negative predictive value) for two assumed prevalence rates: one of 0.04 for the general population, originating from the situations in Wuhan, China^21^ and the Netherlands^22^; and another of 0.5 for a high-risk population.^7^ For a prevalence rate of 0.5, both the PPV and NPV of S2–78 IgG were similar to those of S1 IgG. However, for a prevalence rate of 0.04, the PPV was only 54.7%, whereas the NPV was extremely high, suggesting that the S2–78 antibody detection may effectively exclude negative cases and generate a high false positive detection rate at a low prevalence rate. Nonetheless, for a low prevalence rate, it might be acceptable to perform additional tests by using other antigens to improve the overall performance because the number of real positives was very low.

**Table 2:** Overall performance of S1 and S2–78 for diagnosis

	S1		S2–78	
	Positive	Negative	Positive	Negative
COVID-19	707	22	696	33
Control	2	540	18	524
Specificity (95% CI)	99.6% (98.7–100%)		96.7% (94.8–98.0%)	
Sensitivity (95% CI)	97% (95.5–98.1%)		95.5% (93.7–96.9%)	
Accuracy (95% CI)	98.1% (97.2–98.8%)		96% (94.8–97%)	
Prevalence				
PPV				
0.04	91.0%		54.7%	
0.5	99.6%		97.5%	
NPV				
0.04	99.9%		99.8%	
0.5	97.1%		95.6%	

*PPV* positive predictive value, *NPV* negative predictive value

**Fig. 3:**
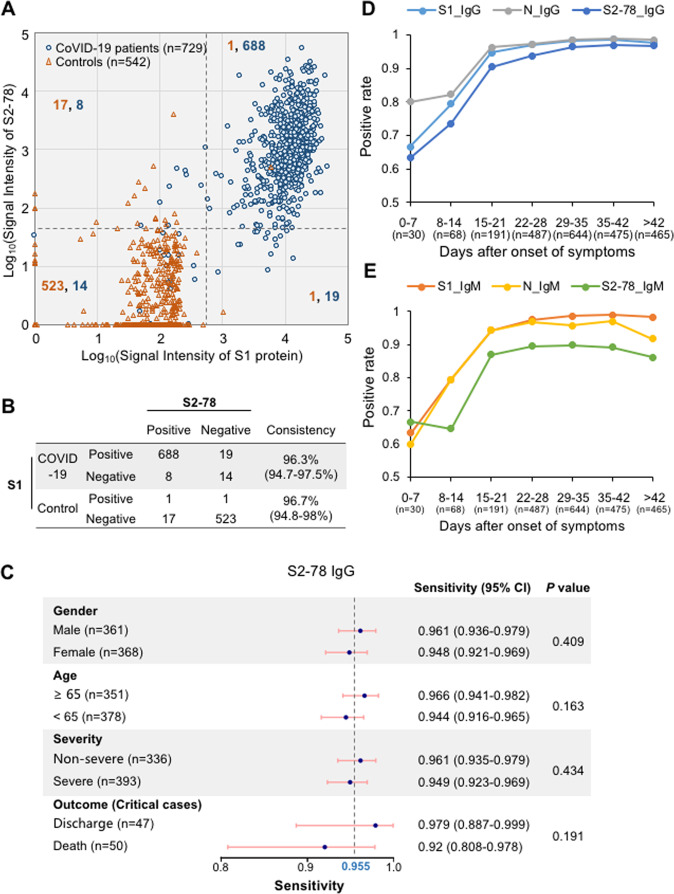
S2–78 (aa 1148–1159) IgG for diagnosis of COVID-19. **A** Scatter plots of serum samples from COVID-19 patients (blue dots) and controls (orange triangle) for S1 IgG vs. S2–78 IgG. The gray lines indicate the cutoff values according to the optimal Youden index based on the ROC curve. The orange and blue numbers indicate the sample counts of controls and patients in each quadrant, respectively. **B** Consistency between S1 protein and S2–78. Consistency values are provided with 95% CIs. **C** Forest plot of the sensitivities of S2–78 IgG among subgroups according to age, sex, severity and outcome. Dots indicate sensitivities; error bars indicate the 95% CI. The exact values are also provided. *P* values were calculated with a *χ*^2^ test. The dashed line (0.955) indicates the overall sensitivity for all patients. **D**, **E** Graph of positive rates of IgG (**D**) or IgM (**E**) against the S1, N proteins and S2-78 versus days after symptom onset in 2360 serum samples from 784 patients

We next investigated the consistency between S1 IgG and S2–78 IgG (Fig. 3A, B). The overall consistencies for the COVID-19 group and control group were 96.3% and 96.7%, respectively. Interestingly, eight samples from COVID-19 patients were negative for S1 IgG but positive for S2–78 IgG, suggesting the diagnostic value of combining S2–78 and S1. It is known that the immune response may correlate with some key clinical parameters, such as sex, disease severity, age and final outcome.^5,9^ To evaluate whether the positive rate of S2–78 IgG is associated with these clinical parameters, we analyzed detection sensitivities among subgroups, i.e., male vs. female, age ≥60 vs. <60, severe vs. nonsevere cases and survivors vs. nonsurvivors with critical diseases. Similar to what we observed for S1 IgG (Supplementary Fig. S2a), no significant difference between any of these subgroups was observed.

Virus-specific antibodies persistently increase and usually undergo seroconversion within one or two weeks after symptom onset, and the proportion of patients with positive antibodies reaches ~100% after two or three weeks.^23,24^ To examine whether S2–78 IgG is suitable for detecting dynamic changes in the antibody response, we analyzed positive rates at different time points after the onset of symptoms. As expected, the rate of S2–78 IgG positivity continuously increased and reached a plateau at approximately three weeks after onset, which was similar to what we observed for S1 and N IgG (Fig. 3D). Similar trends were also found for S2–78 IgM (Fig. 3E) and IgG antibodies against other peptides (Supplementary Fig. S2b). These observations suggest that antibodies against S2–78 and other peptides may be applied for monitoring virus-specific antibody dynamics.

### The diagnostic performance of S2–78 (aa 1148–1159) IgG is comparable to that of S1 IgG for asymptomatic infections

**Fig. 4:**
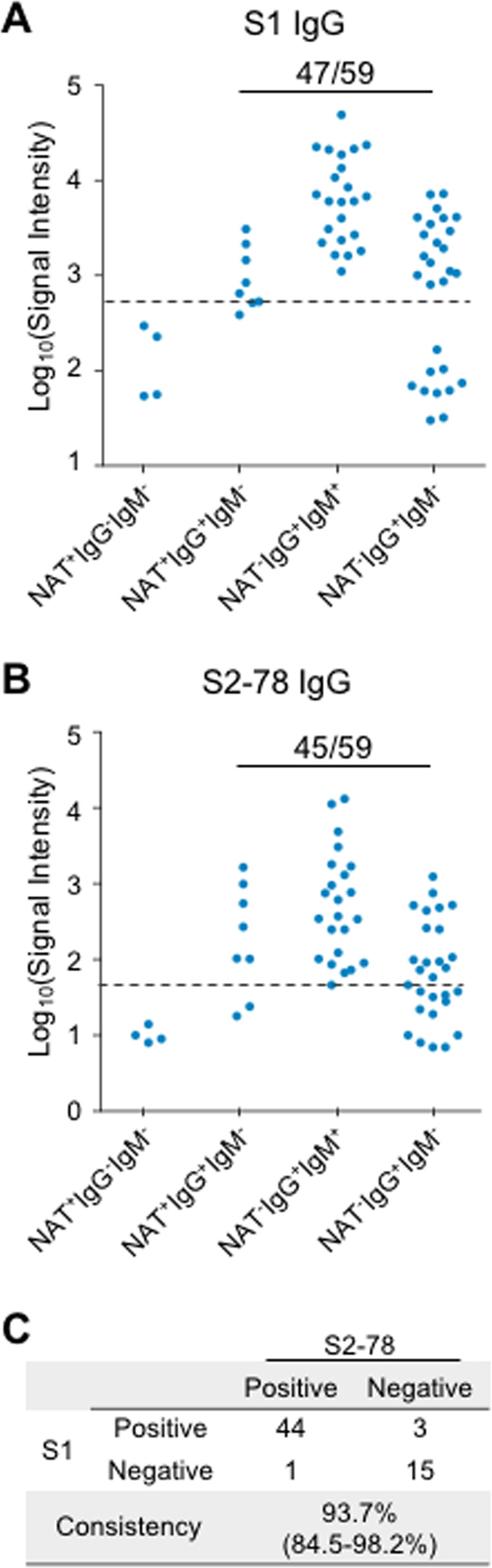
S2–78 (aa 1148–1159) IgG for the diagnosis of asymptomatic patients. **A, B** Levels of S1 IgG and S2–78 IgG in asymptomatic patient groups divided by positive or negative results for three tests, i.e., NAT (nucleic acid test) and SARS-CoV-2 virus-specific IgG and IgM antibodies. Dashed lines indicate cutoff values. The number of positive samples as well as the total number of samples of IgG + (IgG positive) groups are presented for S1 IgG and S2–78 IgG. **C** Consistency between S1 IgG and S2–78 IgG

Monitoring asymptomatic infections is essential to control SARS-CoV-2 infection. It is thought that a lack of symptoms usually indicates a weak immune response.^25^ To test whether S2–78 IgG can be used for the detection of asymptomatic infection, we analyzed 63 asymptomatic cases (Cohort 4, Table 1) defined as positive either by the NAT or antibody test conducted with a commercial assay (see “Methods“) but without obvious symptoms. Four subgroups were divided according to whether they were positive for NAT, IgG and IgM,^26^ whereas there were no cases of IgG- IgM+. For S1 IgG and S2–78 IgG, all samples (*n* = 4) of the IgG^-^ group were negative; of the 59 IgG^+^ cases of asymptomatic infection, 47 and 45 were positive for S1 IgG and S2–78 IgG, respectively (Fig. 4A, B). The consistency between S1 IgG and S2–78 IgG was also high (93.7%). The contradictory results in the IgG^+^ group between our data and that from the commercial assay may be due to differences in the antigens used and the low response levels for many of the inconsistent samples (Supplementary Fig. S3). Recombinant S and N proteins are used for commercial assays, and slightly lower specificity is common.^7^ Overall, these results demonstrate the diagnostic and screening value of S2–78 (aa 1148–1159) IgG for asymptomatic infection.

### The diagnostic value of S2–78 (aa 1148–1159) IgG was validated by ELISA

**Fig. 5:**
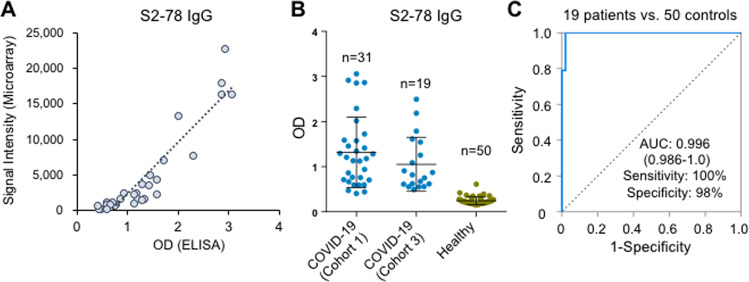
ELISA validation of S2–78 (aa 1148–1159) in Cohort 3. **A** Correlation of signals of S2–78 IgG responses between the peptide microarray and ELISA in Cohort 1 (*n* = 31). **B** Levels of S2–78 IgG in samples from COVID-19 patients either from Cohort 1 (*n* = 31) or Cohort 3 (*n* = 19) and healthy controls (*n* = 50). **C** ROC curve for S2–78 IgG for discrimination of 19 COVID-19 patients (Cohort 3) and 50 healthy controls

ELISA (enzyme-linked immunosorbent assay) is commonly used for commercial SARS-CoV-2 antibody assays.^8,27^ To verify the efficacy and applicability of S2–78 IgG, we accordingly established an ELISA. First, to assess consistency between the peptide microarray and ELISA, we randomly selected 31 sera from COVID-19 patients in Cohort 1 and tested them by ELISA. High consistency was achieved, with a Pearson correlation of 0.926 (Fig. 5A), demonstrating the validity of the microarray results. To further validate the performance of S2–78 IgG, we screened another independent cohort of samples collected from a different medical center (Cohort 3, Table 1). As expected, high performance of S2–78 IgG for specific detection of COVID-19 was achieved by ELISA (Fig. 5B, C).

### A combination of SARS-CoV-2-specific peptides for the detection of COVID-19

**Fig. 6:**
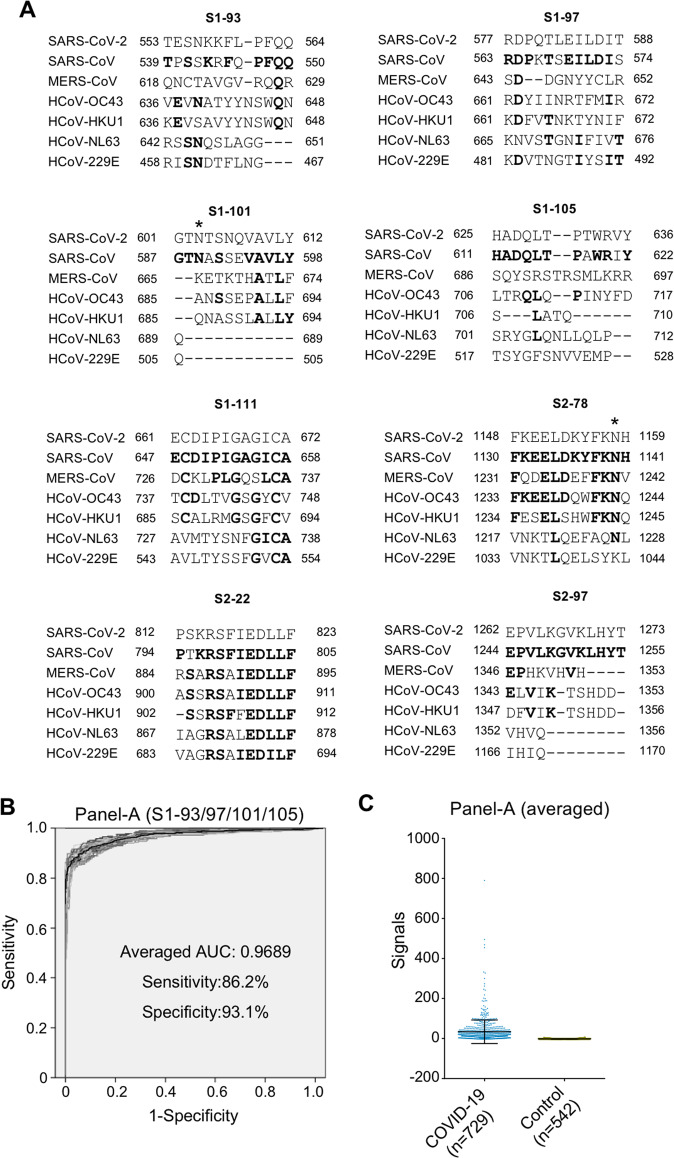
Discrimination of related coronaviruses with a combination of peptides. **A** Homology analysis of significant peptides in SARS-CoV-2, SARS-CoV, MERS-CoV and the other four human coronaviruses. The line at each position indicates a gap. Bold letters indicate positions with the same amino acids as in SARS-CoV-2. The homology analysis was performed with Clustal Omega (https://www.ebi.ac.uk/Tools/msa/clustalo/). The asterisk indicates the glycosylation site in SARS-CoV-2. **B** ROCs of Panel A (S1–93, S1–97, S1–101, and S1–105). One hundred runs of computational cross-validation were performed, and 100 ROC curves were generated (gray). Averaged ROC curves (black) were generated by averaging the 100 ROC curves. **C** Signals of Panel A (S1–93, 97, 101, and 105) in COVID-19 patients and control groups

Because of the highly homologous genomes among human coronaviruses, one unavoidable risk for antibody-based diagnosis of SARS-CoV-2 infection is possible cross-reactivity with other coronaviruses, especially coronaviruses that cause the common cold, i.e., HCoV-OC43, HKU1, NL63 and 229E. The protein sequences of the S protein among these viruses show high similarity, and the selection of S protein sections that are unique to SARS-CoV-2 is important for diagnosis.^11,13^ To investigate whether the identified peptides are specific to SARS-CoV-2, we performed homology analysis among SARS-CoV-2 and 6 other coronaviruses (Fig. 6A). High homology was observed for S2–78 (aa 1148–1159), and S2–22 (aa 812–823), suggesting potential cross-reactivity with other human coronaviruses. In contrast, the other peptides, i.e., S1–93 (aa 553–564), S1–97 (aa 577–588), S1–101 (aa 601–612), S1–105 (aa 625–636), S1–111 (aa 661–672), and S2–97, of SARS-CoV-2 exhibit low similarity with other coronaviruses, particularly the four coronaviruses that cause the common cold in humans, suggesting that they may be applied for specific detection of SARS-CoV-2 infection. To further improve performance, we attempted to construct a panel by combining several peptides. After initial screening by bivariate regression analysis, we found that among these SARS-CoV-2-specific peptides, S1–93 (aa 553–564), S1–97 (aa 577–588), S1–101 (aa 601–612), and S1–105 (aa 625–636) may be combined as a panel (Panel A) that enhances performance. Ideally, it would be best to validate the diagnostic power of these peptides by using independent sample cohorts. However, it is very difficult to collect additional COVID-19 samples at present because of the very strict regulations for sample handling and the lack of COVID-19 patients in China. To avoid overfitting, we decided to perform 100 runs of computational cross-validation using the large-sample cohort that we analyzed with the peptide microarray, followed by a protocol that we established previously.^28^ For each run, half of the patients and half of the controls were randomly selected as the training cohort; the rest of the samples were used as the test cohort. The result for each of these tests was presented as a ROC curve, and an average ROC curve was ultimately generated (Fig. 6C). Very similar curves were built for all cross-validations, demonstrating the robustness of this panel. According to the average curve, the sensitivity and specificity of **a** were 86.2% and 93.1%, respectively (Fig. 6B, C).

## Discussion

In this study, we took advantage of a peptide microarray with full S protein coverage^19,29^ and analyzed 2,434 sera from 858 COVID-19 patients, 63 asymptomatic patients and 610 controls collected from multiple medical centers. We identified eight 12-mer peptides (“significant” peptides) that exhibit high diagnostic value as antigens for detecting SARS-CoV-2-specific IgG or IgM. Among the “significant” peptides, S2–78 (aa 1148–1159) has a diagnostic performance comparable to that of the S1 protein for the detection of COVID-19 and asymptomatic infection, suggesting that S2–78 has the potential to be used in serological prevalence investigations.

Serological tests play an important role in the diagnosis and monitoring of COVID-19. Recombinant proteins, particularly S1, are one of the key reagents used to produce immunoassays for detecting SARS-CoV-2 IgG, IgM or IgA. However, expressing S1 protein in the correct conformation is difficult, and in some cases, the antibodies that recognize the membrane S protein are unable to bind recombinant S.^30^ Moreover, variability in the proteins obtained from different manufacturers and even different batches from the same manufacturer may result in high variation. In addition, high cost and insufficient capacity to produce enough high-quality recombinant S1 protein limit accessibility to the immunoassay in poor or remote regions around the world. Alternatively, peptide-based immunoassays provide a superior choice compared to S1 protein assays. The reasons for this are as follows. (1) Peptide synthesis can be easily scaled up when required. Indeed, a large amount of peptide can be easily synthesized within a very short period of time; if necessary, the peptide can be synthesized in a GMP facility. (2) The consistency and purity resulting from peptide synthesis is high, and there is almost no batch-to-batch variation. (3) The peptide is very stable, and as a reagent, it can be easily stored and transported. (4) The cost of the peptide is approximately 100–1000-fold lower than that of the S1 protein.

We identified S2–78 (aa 1148–1159) as a candidate of high diagnostic value. In fact, our previous study and other studies also identified S2–78 or related regions as linear epitopes with high response frequency^16,18,19^; however, the diagnostic power of S2–78 was not revealed by these studies, likely due to the limited sample size used for the initial screening or the difference in platform.^15^ By examining large cohorts of COVID-19 patients and a variety of controls, we comprehensively verified S2–78 IgG as a good candidate for COVID-19 diagnosis, with specificity and sensitivity comparable to those of S1 IgG. As expected, we found that the overall consistency of detection with S2–78 IgG and S1 IgG was high, suggesting that S2–78 has the potential to replace the S1 protein. Notably, the signal intensity level of S2–78 IgG was lower than that of S1 IgG, which is reasonable since there are multiple sites on the S1 protein that can be recognized by antibodies in COVID-19 sera. Nevertheless, because it is difficult to ensure extremely high purity when purifying recombinant proteins, the background S1 IgG level in the control group was higher than the peptide level. These results indicate that the sensitivity of S2–78 IgG might be further evaluated when increasing the antigen concentration or adopting more sensitive platforms for detection, such as electrochemical platforms or single-molecule detection technologies.^31,32^ In fact, we also performed S1-based ELISAs using a commercial kit with the same set of samples, and the overall performance of the S2–78 IgG-based ELISA was comparable to that of the commercial kits (data not shown).

Immunoassays are the main tool used to assess the extent of virus circulation in populations and the likelihood of protection against reinfection by screening populations to identify infected individuals without clinical symptoms.^7,12,21^ We verified that S2–78 (aa 1148–1159) IgG can be applied to detect asymptomatic infections, with a sensitivity and specificity comparable to that of S1 IgG, even though asymptomatic individuals are thought to have weaker immune responses.^25^ We assessed the PPV and NPV for two prevalence rates, 0.04 and 0.5; for the former, the PPV was 54.7%, and the NPV was 99.8%, indicating a high false positive rate but effective and accurate exclusion of negative cases. Due to the small number of positive cases under this circumstance, one possible solution is to retest suspected samples with additional assays. Moreover, to meet the requirements of population-wide application, the performance of S2–78 might be improved by optimizing the parameters or adopting other platforms.

The S protein shares high sequence similarity with other seasonal circulating human coronaviruses. Theoretically, cross-reactivity may exist when S/S1 is applied as an antigen for immunological tests, thus leading to false positives. It is therefore necessary to pinpoint specific regions/sites of the SARS-CoV-2 S protein to eliminate potential cross-reactivity. Overall, selection of peptides with high antibody responses and low sequence similarities with other human coronaviruses will improve diagnostic performance. Among the identified peptides, except for S2–78 (aa 1148–1159) and S2–22 (aa 812–823), the peptides are very distinct from those in the four circulating human coronaviruses, suggesting that they may serve as specific antigens to eliminate potential cross-activity. Moreover, we constructed and validated Panel A (S1–93, S1–97, S1–105, and S1–111) based on these SARS-CoV-2 peptides, achieving a sensitivity of 93.1% and a specificity of 86.2%. Hence, Panel A may be applied to avoid potential cross-reactivity with other human coronaviruses and achieve better specificity than the whole S protein or other peptides.

In summary, we identified and verified eight peptides derived from the S protein that exhibit high diagnostic value. These peptides might be used in different circumstances alone or in combination as candidates to generate immunoassays for monitoring COVID-19. In comparison to the current protein-based immunoassays, peptide-based assays will be highly affordable and accessible.

## Materials and methods

### Patients and samples

The study was approved by the Ethical Committee of Tongji Hospital, Tongji Medical College, Huazhong University of Science and Technology, Wuhan, China (ITJ-C20200128), the Institutional Ethics Review Committee of Foshan Fourth Hospital, Foshan, China (202005) and the Ethical Committee of The Fifth Affiliated Hospital of Sun Yat-sen University, Zhuhai, China (K14-2). Written informed consent was obtained from all participants enrolled in this study. COVID-19 patients were hospitalized and received treatment in multiple medical centers during the period from 25 January 2020 to 28 April 2020. Sera from the control group of healthy donors, lung cancer patients, and patients with autoimmune diseases were collected from Ruijin Hospital, Shanghai, China or Tongren Hospital, Shanghai, China. The negative reference samples were obtained from the National Institutes for Food and Drug Control. The sera of 63 asymptomatic patients were acquired from Tongji Hospital, Wuhan. IgM and IgG antibodies against the recombinant nucleoprotein and spike protein of SARS-CoV-2 in the sera of patients were detected using a commercial kit (YHLO Biotech, Shenzhen, China). According to the instructions of the kit, an antibody level ≥10 AU/mL is positive, and a level <10 AU/mL is negative. All samples were stored at −80 °C until use.

### Peptide synthesis and conjugation with BSA

N-terminal amidated peptides were synthesized by GL Biochem, Ltd. (Shanghai, China). Each peptide was individually conjugated with BSA using Sulfo-SMCC (Thermo Fisher Scientific, MA, USA) according to the manufacturer’s instructions. Briefly, BSA was activated by Sulfo-SMCC at a molar ratio of 1:30, followed by dialysis in PBS buffer. The peptide with cysteine was added at a w/w ratio of 1:1 and incubated for 2 h, followed by dialysis in PBS to remove the free peptides. A few conjugates were randomly selected for examination by SDS-PAGE. For the biotin-BSA-peptide conjugates, before conjugation, BSA was labeled with biotin by using NHS-LC-Biotin reagent (Thermo Fisher Scientific, MA, USA) at a molar ratio of 1:5 and then activated by Sulfo-SMCC.

### Peptide microarray fabrication

The peptide-BSA conjugates as well as the S1 protein, RBD protein and N protein of SARS-CoV-2, along with negative (BSA) and positive controls (anti-human IgG and IgM antibody), were printed in triplicate on PATH substrate slides (Grace Bio-Labs, Oregon, USA) to generate identical arrays in a 1 × 7 (for the peptide microarray with three concentrations) or 2 × 7 subarray format (for the peptide microarray with one concentration) using a Super Marathon printer (Arrayjet, UK). The microarrays were stored at −80 °C until use.

### Microarray-based serum analysis

A 7- or 14-chamber rubber gasket was mounted onto each slide to create individual chambers for the 7- or 14-chamber identical subarrays. The microarray was used for serum profiling as described previously, with minor modifications.^33^ Briefly, the arrays stored at −80 °C were warmed to room temperature and then incubated in blocking buffer (3% BSA in 1× PBS buffer with 0.1% Tween 20) for 3 h. A total of 400 μL for the 7-subarray format or 200 μL for the 14-subarray format of diluted sera or antibodies was incubated with each subarray for 2 h. For most samples, sera were diluted at 1:200; free peptides were added at a concentration of 0.25 mg/mL. For the enriched antibodies, 0.1–0.5 μg antibodies were included in 200 μL incubation buffer. The arrays were washed with 1× PBST, and the bound antibodies were detected by incubating the arrays with Cy3-conjugated goat anti-human IgG and Alexa Fluor 647-conjugated donkey anti-human IgM (Jackson ImmunoResearch, PA, USA), which were diluted 1:1000 in 1× PBST. The incubation was carried out at room temperature for 1 h. The microarrays were then washed with 1× PBST, dried by centrifugation at room temperature and scanned using a LuxScan 10K-A (CapitalBio Corporation, Beijing, China) with the parameters set as 95% laser power/PMT 550 and 95% laser power/PMT 480 for IgM and IgG, respectively. The fluorescence intensity was evaluated using GenePix Pro 6.0 software (Molecular Devices, CA, USA).

### ELISA

Briefly, 96-well microplates with a high-binding polystyrene surface (Corning, New York, USA) were coated with 100 μL BSA-conjugated peptide (S2–78, aa 1148–1159) at 100 μg/mL and incubated overnight at 4 °C. The plates were washed once with PBST buffer (PBS buffer with 0.1% Tween 20), blocked with 3% BSA (bovine serum albumin) for 1 h at room temperature, and washed once with PBST. Sera were diluted at 1:50 in PBST buffer with 1% BSA, 1% FBS and 3% horse serum; 100 μL of the preparation was loaded into each well, and incubation was carried out at 37 °C for 1.5 h. After six washes with PBST, the secondary antibody, i.e., anti-human IgG-peroxidase (Sangon Biotech, Shanghai, China), was diluted to 1:10000 and incubated at 37 °C for 1 h, which was followed by eight washes. The tetramethylbenzidine substrate (Sigma-Aldrich, Missouri, USA) was then added and incubated for 20 min. Finally, 50 μL sulfuric acid (2 M) was added to stop the reaction. Optical density was measured at 450 nm using a Behring EL311 ELISA microplate reader (Dade Behring Marburg Gmbh, Berlin, Germany). The assays were repeated twice for each sample.

### Data analysis for the peptide microarray

For each spot, the signal intensity was defined as the foreground minus the background. The signal intensities of triplicate spots for each peptide or protein were averaged. For the samples from Cohorts 2 and 3, normalization among the microarray slides was performed. For each slide, block #14 was incubated with the positive reference sample, which was generated by pooling 50 randomly selected sera from COVID-19 patients. For each slide, the data generated from the positive reference sample were subjected to linear regression, and the data from other samples were subjected to linear normalization according to the function. GraphPad 6.0 was used to generate ROC curves and calculate AUC values. Computational cross-validation was performed as described previously.^28^ Briefly, to validate Panel A, for each run, half of the patients and half of the controls were randomly selected as the training cohort to perform bivariate regression to generate a linear regression function. The other half of the samples were tested with this function to acquire a ROC, and a value for each sample was then obtained with the function. The average of the ROC curve values was calculated after 100 runs of computational cross-validation.

## Supplementary information

Supplementary information

## Data Availability

The peptide microarray data generated in this study have been deposited in Protein Microarray Database (http://www.proteinmicroarray.cn) under the accession numbers PMDE242 and PMDE244. Additional data related to this study may be requested from the authors.

## References

[1] Zhou P (2020). A pneumonia outbreak associated with a new coronavirus of probable bat origin. Nature.

[2] Wu F (2020). A new coronavirus associated with human respiratory disease in China. Nature.

[3] Dong E, Du H, Gardner L (2020). An interactive web-based dashboard to track COVID-19 in real time. Lancet Infect. Dis..

[4] Lee CYP, Lin RTP, Renia L, Ng LFP (2020). Serological approaches for COVID-19: epidemiologic perspective on surveillance and control. Front. Immunol..

[5] Long QX (2020). Antibody responses to SARS-CoV-2 in patients with COVID-19. Nat. Med..

[6] Peeling RW (2020). Serology testing in the COVID-19 pandemic response. Lancet Infect. Dis..

[7] GeurtsvanKessel CH (2020). An evaluation of COVID-19 serological assays informs future diagnostics and exposure assessment. Nat. Commun..

[8] Yan Y, Chang L, Wang L (2020). Laboratory testing of SARS-CoV, MERS-CoV, and SARS-CoV-2 (2019-nCoV): current status, challenges, and countermeasures. Rev. Med. Virol..

[9] Jiang H (2020). SARS-CoV-2 proteome microarray for global profiling of COVID-19 specific IgG and IgM responses. Nat. Commun..

[10] Vabret N (2020). Immunology of COVID-19: current state of the science. Immunity.

[11] Petherick A (2020). Developing antibody tests for SARS-CoV-2. Lancet.

[12] Lisboa Bastos M (2020). Diagnostic accuracy of serological tests for covid-19: systematic review and meta-analysis. BMJ.

[13] Hicks, J. et al. Serologic cross-reactivity of SARS-CoV-2 with endemic and seasonal Betacoronaviruses. *medRxiv*10.1101/2020.06.22.20137695 (2020). 10.1007/s10875-021-00997-6 PMC7962425 33725211

[14] Wang H (2020). SARS-CoV-2 proteome microarray for mapping COVID-19 antibody interactions at amino acid resolution. ACS Cent Sci..

[15] Poh CM (2020). Two linear epitopes on the SARS-CoV-2 spike protein that elicit neutralising antibodies in COVID-19 patients. Nat. Commun..

[16] Zamecnik CR (2020). ReScan, a multiplex diagnostic pipeline, Pans Human Sera for SARS-CoV-2 antigens. Cell Rep Med..

[17] Farrera-Soler L (2020). Identification of immunodominant linear epitopes from SARS-CoV-2 patient plasma. PLoS ONE.

[18] Mishra, N. et al. Immunoreactive peptide maps of SARS-CoV-2 and other human coronaviruses. *bioRxiv*10.1101/2020.08.13.249953 (2020).

[19] Li Y (2020). Linear epitopes of SARS-CoV-2 spike protein elicit neutralizing antibodies in COVID-19 patients. Cell Mol. Immunol..

[20] WHO. WHO Laboratory testing for 2019 novel coronavirus (2019-nCoV) in suspected human cases. 2020.

[21] Xu X (2020). Seroprevalence of immunoglobulin M and G antibodies against SARS-CoV-2 in China. Nat. Med..

[22] Slot, E. et al. Herd immunity is not a realistic exit strategy during a COVID-19 outbreak. *Nat. Res.*10.21203/rs.3.rs-25862/v1 (2020).

[23] Sikora, M. et al. Map of SARS-CoV-2 spike epitopes not shielded by glycans. *bioRxiv*10.1101/2020.07.03.186825 (2020).

[24] Xiang, F. et al. Antibody detection and dynamic characteristics in patients with COVID-19. *Clin. Infect. Dis.*10.1093/cid/ciaa461 (2020). 10.1093/cid/ciaa461 PMC7188146 32306047

[25] Long QX (2020). Clinical and immunological assessment of asymptomatic SARS-CoV-2 infections. Nat. Med..

[26] Lei, Q. et al. Antibody dynamics to SARS-CoV-2 in asymptomatic COVID-19 infections. *Allergy*10.1111/all.14622 (2020). 10.1111/all.14622 PMC7675426 33040337

[27] Zhang, X. et al. Proteome-wide analysis of differentially-expressed SARS-CoV-2 antibodies in early COVID-19 infection. *medRxiv*10.1101/2020.04.14.20064535 (2020).

[28] Yang L (2016). Identification of serum biomarkers for gastric cancer diagnosis using a human proteome microarray. Mol. Cell Proteom..

[29] Li, Y. et al. Linear epitope landscape of SARS-CoV-2 Spike protein constructed from 1051 COVID-19 patients. *medRxiv*10.1101/2020.07.13.20152587 (2020). 10.1016/j.celrep.2021.108915 PMC7953450 33761319

[30] Wan J (2020). Human IgG cell neutralizing monoclonal antibodies block SARS- CoV-2 infection. Cell Rep..

[31] Croop B, Zhang C, Lim Y, Gelfand RM, Han KY (2019). Recent advancement of light-based single-molecule approaches for studying biomolecules. Wiley Interdiscip. Rev. Syst. Biol. Med..

[32] McEachern F, Harvey E, Merle G (2020). Emerging technologies for the electrochemical detection of bacteria. Biotechnol. J..

[33] Li Y (2020). Longitudinal serum autoantibody repertoire profiling identifies surgery-associated biomarkers in lung adenocarcinoma. EBioMedicine.

