# Supplementary material for: Systematic evaluation of IgG responses to SARS-CoV-2 spike protein-derived peptides for monitoring COVID-19 patients
Source: Cell Mol Immunol. 2021 Jan 22;18(3):621–31. doi: 10.1038/s41423-020-00612-5 (PMC7821179; doi:10.1038/s41423-020-00612-5)
Supplement: Supplementary file 1 — Supplementary information [file 41423_2020_612_MOESM1_ESM.pdf]

Supplementary Information

Systematic evaluation IgG response of SARS-CoV-2 spike protein derived peptides for monitoring of COVID-19 patients

Li, et al

Supplementary Fig. 1. Evaluation of the significant peptides by Cohort 2 –other peptides.
Supplementary Fig. 2. Performance of peptides and S1 for diagnosis in subgroups.
Supplementary Fig.3. Consistence between the commercial assay and our platform based on S or N protein.
Supplementary Table 1 The peptides synthesized in this study.

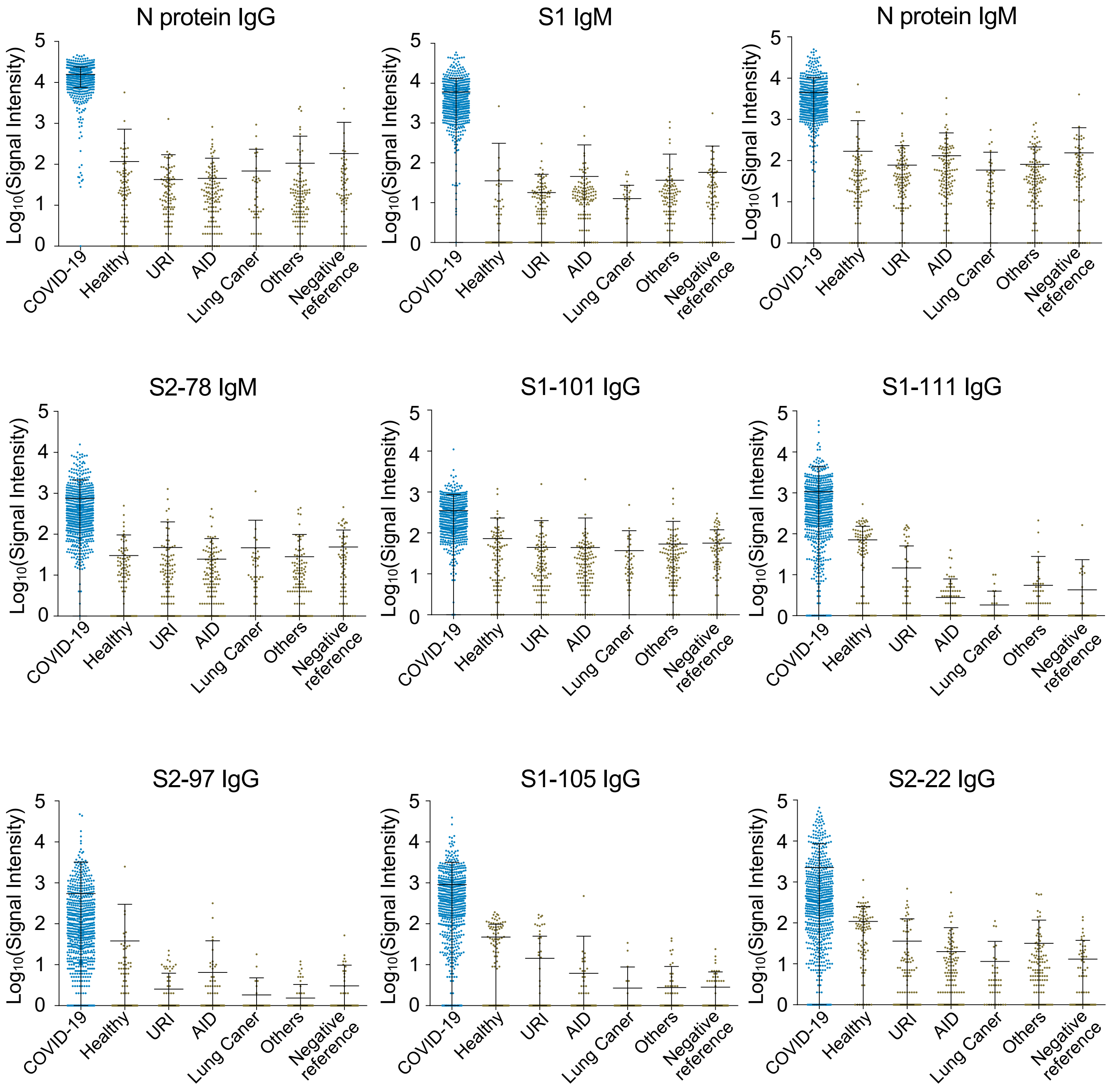

Fig. S1. Evaluation of the significant peptides by Cohort 2 – other peptides. Signal levels of the antibodies against the indicated peptides in COVID-19 patients (n=729), Healthy controls (n=92), upper respiratory infections (URI, n=104), patients with autoimmune diseases (AID, n=120), lung cancer patients (n=41), patients with other diseases (n=112) and negative reference samples (n=73) of Cohort 2.

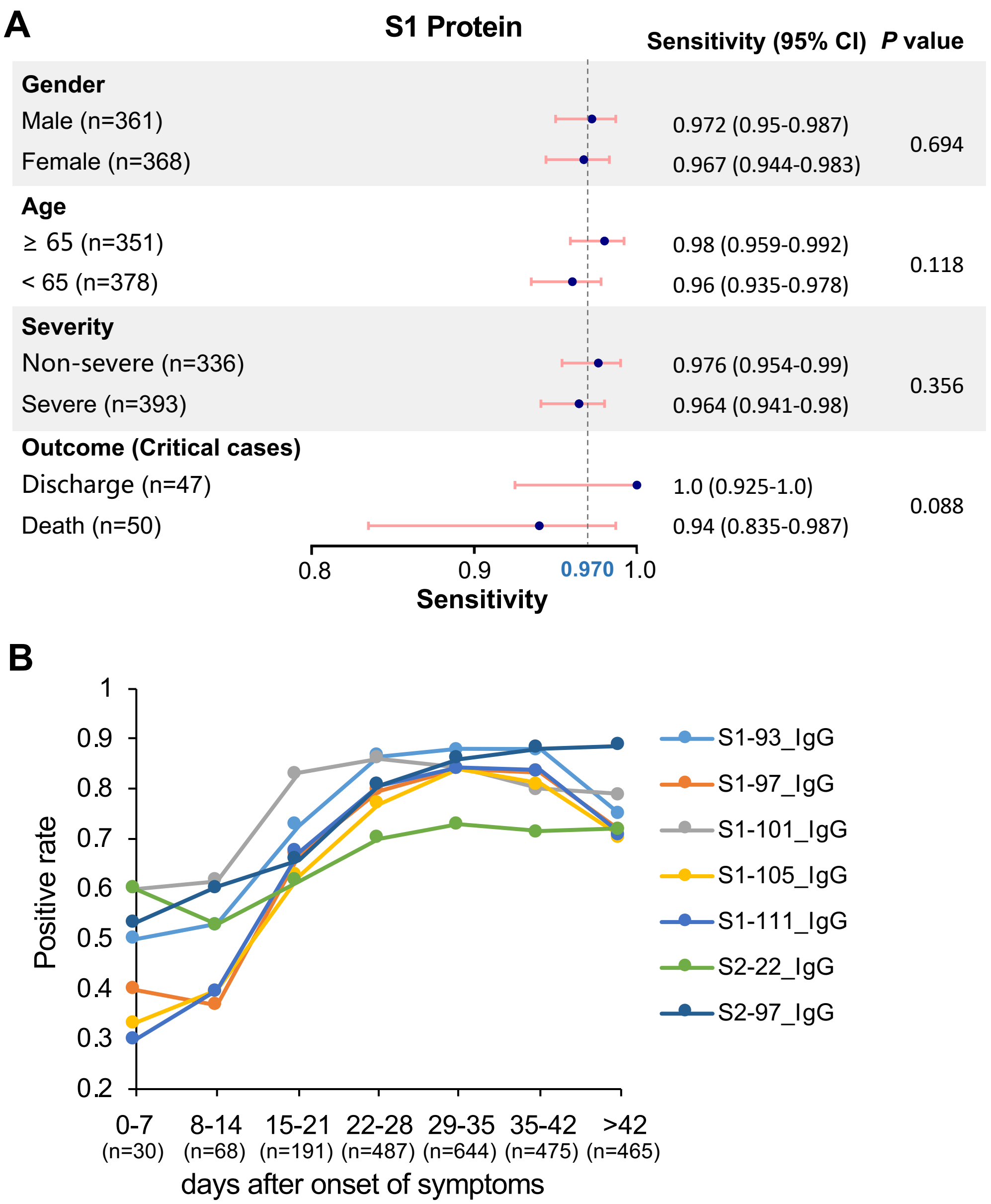

Fig. S2. Performance of peptides and S1 for diagnosis in subgroups. a. forest plot of sensitivities of S1 IgG in different subgroups, i.e., age, gender, severity and outcome. The dots indicate the sensitivities while the error bars indicate the 95% CI. The exact values are also provided. P values were calculated with $\chi 2$ test. The dashed line (0.970) indicates the overall sensitivity of all the patients. b. Graph of positive rates of IgG antibodies against the indicated peptides versus days after symptom onset in 2,360 serum samples from 784 patients.

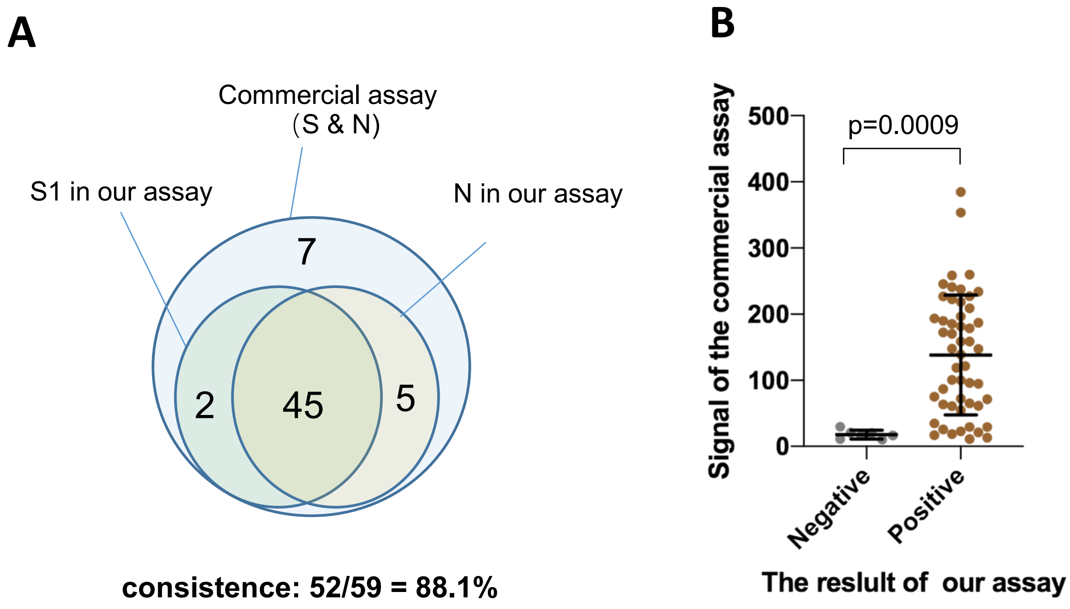

Fig. S3. Consistence between the commercial assay and our platform based on S or N protein. A. the results and comparison of the 59 antibody positive asymptomatic patients. In combination S and N response, 52 positive based on our platform and the consistence with the commercial assay is 88.1% (52/59). **B.** Comparison of signal level of the commercial assay between the two groups divided based on our data, either false negative or true positive.

Table S1. The peptides synthesized in this study

NO.	Peptide ID	Sart Position	Amino acid sequence	End Position	Note
1	S1-1	1	MFVFLVLLPLVS	12	N/A
2	S1-2	7	LLPLVSSQCVNL	18	
3	S1-3	13	SQCVNLTTRTQL	24	N/A
4	S1-4	19	TTRTQLPPAYTN	30	
5	S1-5	25	PPAYTNSFTRGV	36	
6	S1-6	31	SFTRGVYYPDKV	42	
7	S1-7	37	YYPDKVFRSSVL	48	
8	S1-8	43	FRSSVLHSTQDL	54	
9	S1-9	49	HSTQDLFLPFFS	60	
10	S1-10	55	FLPFFSNVTWFH	66	N/A
11	S1-11	61	NVTWFHAIHVSG	72	
12	S1-12	67	AIHVSGTNGTKR	78	
13	S1-13	73	TNGTKRFDNPVL	84	
14	S1-14	79	FDNPVLPFNDGV	90	
15	S1-15	85	PFNDGVYFASTE	96	
16	S1-16	91	YFASTEKSNIIR	102	
17	S1-17	97	KSNIIRGWIFGT	108	
18	S1-18	103	GWIFGTTLDSKT	114	
19	S1-19	109	TLDSKTQSLLIV	120	
20	S1-20	115	QSLLIVNNATNV	126	
21	S1-21	121	NNATNVVIKVCE	132	
22	S1-22	127	VIKVCEFQFCND	138	
23	S1-23	133	FQFCNDPFLGVY	144	
24	S1-24	139	PFLGVYYHKNNK	150	
25	S1-25	145	YHKNNKSWMESE	156	
26	S1-26	151	SWMESEFRVYSS	162	
27	S1-27	157	FRVYSSANNCTF	168	
28	S1-28	163	ANNCTFEYVSQP	174	
29	S1-29	169	EYVSQPFLMDLE	180	
30	S1-30	175	FLMDLEGKQGNF	186	
31	S1-31	181	GKQGNFKNLREF	192	
32	S1-32	187	KNLREFVFKNID	198	N/A
33	S1-33	193	VFKNIDGYFKIY	204	N/A
34	S1-34	199	GYFKIYSKHTPI	210	
35	S1-35	205	SKHTPINLVRDL	216	N/A
36	S1-36	211	NLVRDLPQGFSA	222	
37	S1-37	217	PQGFSALEPLVD	228	N/A
38	S1-38	223	LEPLVDLPIGIN	234	
39	S1-39	229	LPIGINITRFQT	240	
40	S1-40	235	ITRFQTLLALHR	246	N/A
41	S1-41	241	LLALHRSYLTPG	252	
42	S1-42	247	SYLTPGDSSSGW	258	
43	S1-43	253	DSSSGWTAGAAA	264	
44	S1-44	259	TAGAAAYYVGYL	270	
45	S1-45	265	YYVGYLQPRTFL	276	
46	S1-46	271	QPRTFLLKYNEN	282	N/A
47	S1-47	277	LKYNENGTITDA	288	
48	S1-48	283	GTITDAVDCALD	294	
49	S1-49	289	VDCALDPLSETK	300	
50	S1-50	295	PLSETKCTLKSF	306	
51	S1-51	301	CTLKSFTVEKGI	312	
52	S1-52	307	TVEKGIYQTSNF	318	
53	S1-53	313	YQTSNFRVQPTE	324	
54	S1-54	319	RVQPTESIVRFP	330	
55	S1-55	325	SIVRFPNITNLC	336	
56	S1-56	331	NITNLCPFGEVF	342	
57	S1-57	337	PFGEVFNATRFA	348	
58	S1-58	343	NATRFASVYAWN	354	
59	S1-59	349	SVYAWNRKRISN	360	
60	S1-60	355	RKRISNCVADYS	366	
61	S1-61	361	CVADYSVLYNSA	372	N/A
62	S1-62	367	VLYNSASFSTFK	378	
63	S1-63	373	SFSTFKCYGVSP	384	
64	S1-64	379	CYGVSPTKLNDL	390	

65	S1-65	385	TKLNDLCFTNVY	396	
66	S1-66	391	CFTNVYADSFVI	402	
67	S1-67	397	ADSFVIRGDEVR	408	
68	S1-68	403	RGDEVRQIAPGQ	414	
69	S1-69	409	QIAPGQTGKIAD	420	
70	S1-70	415	TGKIADYNYKLP	426	
71	S1-71	421	YNYKLPDDFTGC	432	
72	S1-72	427	DDFTGCVIAWNS	438	
73	S1-73	433	VIAWNSNNLDSK	444	
74	S1-74	439	NNLDSKVGGNYN	450	
75	S1-75	445	VGGNYNYLYRLF	456	N/A
76	S1-76	451	YLYRLFRKSNLK	462	
77	S1-77	457	RKSNLKPFERDI	468	
78	S1-78	463	PFERDISTEIYQ	474	
79	S1-79	469	STEIYQAGSTPC	480	
80	S1-80	475	AGSTPCNGVEGF	486	
81	S1-81	481	NGVEGFNCYFPL	492	
82	S1-82	487	NCYFPLQSYGFQ	498	
83	S1-83	493	QSYGFQPTNGVG	504	
84	S1-84	499	PTNGVGYQPYRV	510	
85	S1-85	505	YQPYRVVVLSFE	516	
86	S1-86	511	VVLSFELLHAPA	522	
87	S1-87	517	LLHAPATVCGPK	528	
88	S1-88	523	TVCGPKKSTNLV	534	
89	S1-89	529	KSTNLVKNKCVN	540	
90	S1-90	535	KNKCVNFNFNGL	546	
91	S1-91	541	FNFNGLTGTGVL	552	
92	S1-92	547	TGTGVLTESNKK	558	
93	S1-93	553	TESNKKFLPFQQ	564	
94	S1-94	559	FLPFQQFGRDIA	570	
95	S1-95	565	FGRDIADTTDAV	576	
96	S1-96	571	DTTDAVRDPQTL	582	
97	S1-97	577	RDPQTLEILDIT	588	
98	S1-98	583	EILDITPCSFGG	594	
99	S1-99	589	PCSFGGVSVITP	600	
100	S1-100	595	VSVITPGTNTSN	606	
101	S1-101	601	GTNTSNQVAVLY	612	
102	S1-102	607	QVAVLYQDVNCT	618	
103	S1-103	613	QDVNCTEVPVAI	624	
104	S1-104	619	EVPVAIHADQLT	630	
105	S1-105	625	HADQLTPTWRVY	636	
106	S1-106	631	PTWRVYSTGSNV	642	
107	S1-107	637	STGSNVFQTRAG	648	N/A
108	S1-108	643	FQTRAGCLIGAE	654	
109	S1-109	649	CLIGAEHVNNSY	660	
110	S1-110	655	HVNNSYECDIPI	666	
111	S1-111	661	ECDIPIGAGICA	672	
112	S1-112	667	GAGICASYQTQT	678	
113	S1-113	673	SYQTQTNSPRRA	684	
114	S1-114	679	NSPRRARGGGGS	685	
115	S2-1	686	SVASQSIIAYTM	697	N/A
116	S2-2	692	IIAYTMSLGAEN	703	N/A
117	S2-3	698	SLGAENSVAYSN	709	
118	S2-4	704	SVAYSNNSIAIP	715	
119	S2-5	710	NSIAIPTNFTIS	721	
120	S2-6	716	TNFTISVTTEIL	727	
121	S2-7	722	VTTEILPVSMTK	733	
122	S2-8	728	PVSMTKTSVDCT	739	
123	S2-9	734	TSVDCTMYICGD	745	
124	S2-10	740	MYICGDSTECSN	751	
125	S2-11	746	STECSNLLLQYG	757	
126	S2-12	752	LLLQYGSFCTQL	763	
127	S2-13	758	SFCTQLNRALTG	769	N/A
128	S2-14	764	NRALTGIAVEQD	775	
129	S2-15	770	IAVEQDKNTQEV	781	
130	S2-16	776	KNTQEVFAQVKQ	787	
131	S2-17	782	FAQVKQIYKTPP	793	

132	S2-18	788	IYKTPPIKDFGG	799	
133	S2-19	794	IKDFGGFNFSQI	805	
134	S2-20	800	FNFSQILPDPSK	811	
135	S2-21	806	LPDPSKPSKRSF	817	
136	S2-22	812	PSKRSFIEDLLF	823	
137	S2-23	818	IEDLLFNKVTLA	829	
138	S2-24	824	NKVTLADAGFIK	835	
139	S2-25	830	DAGFIKQYGDCL	841	
140	S2-26	836	QYGDCLGDIAAR	847	
141	S2-27	842	GDIAARDLICAQ	853	
142	S2-28	848	DLICAQKFNGLT	859	
143	S2-29	854	KFNGLTVLPPLL	865	
144	S2-30	860	VLPPLLTDEMIA	871	
145	S2-31	866	TDEMIAQYTSAL	877	
146	S2-32	872	QYTSALLAGTIT	883	
147	S2-33	878	LAGTITSGWTFG	889	
148	S2-34	884	SGWTFGAGAALQ	895	
149	S2-35	890	AGAALQIPFAMQ	901	
150	S2-36	896	IPFAMQMAYRFN	907	
151	S2-37	902	MAYRFNGIGVTQ	913	
152	S2-38	908	GIGVTQNVLYEN	919	
153	S2-39	914	NVLYENQKLIAN	925	
154	S2-40	920	QKLIANQFNSAI	931	
155	S2-41	926	QFNSAIGKIQDS	937	
156	S2-42	932	GKIQDSLSSTAS	943	
157	S2-43	938	LSSTASALGKLQ	949	
158	S2-44	944	ALGKLQDVVNQN	955	
159	S2-45	950	DVVNQNAQALNT	961	
160	S2-46	956	AQALNTLVKQLS	967	
161	S2-47	962	LVKQLSSNFGAI	973	
162	S2-48	968	SNFGAISSVLND	979	
163	S2-49	974	SSVLNDILSRLD	985	
164	S2-50	980	ILSRLDKVEAEV	991	
165	S2-51	986	KVEAEVQIDRLI	997	
166	S2-52	992	QIDRLITGRLQS	1003	
167	S2-53	998	TGRLQSLQTYVT	1009	
168	S2-54	1004	LQTYVTQQLIRA	1015	
169	S2-55	1010	QQLIRAAEIRAS	1021	
170	S2-56	1016	AEIRASANLAAT	1027	
171	S2-57	1022	ANLAATKMSECV	1033	
172	S2-58	1028	KMSECVLGQSKR	1039	
173	S2-59	1034	LGQSKRVDFCGK	1045	
174	S2-60	1040	VDFCGKGYHLMS	1051	
175	S2-61	1046	GYHLMSFPQSAP	1057	
176	S2-62	1052	FPQSAPHGVVFL	1063	
177	S2-63	1058	HGVVFLHVTYVP	1069	
178	S2-64	1064	HVTYVPAQEKNF	1075	
179	S2-65	1070	AQEKNFTTAPAI	1081	
180	S2-66	1076	TTAPAICHDGKA	1087	
181	S2-67	1082	CHDGKAHFPREG	1093	
182	S2-68	1088	HFPREGVFVSNG	1099	
183	S2-69	1094	VFVSNGTHWFVT	1105	
184	S2-70	1100	THWFVTQRNFYE	1111	
185	S2-71	1106	QRNFYEPQIITT	1117	
186	S2-72	1112	PQIITTDNTFVS	1123	
187	S2-73	1118	DNTFVSGNCDVV	1129	
188	S2-74	1124	GNCDVVIGIVNN	1135	N/A
189	S2-75	1130	IGIVNNTVYDPL	1141	
190	S2-76	1136	TVYDPLQPELDS	1147	
191	S2-77	1142	QPELDSFKEELD	1153	
192	S2-78	1148	FKEELDKYFKNH	1159	
193	S2-79	1154	KYFKNHTSPDVD	1165	
194	S2-80	1160	TSPDVDLGDISG	1171	
195	S2-81	1166	LGDISGINASVV	1177	
196	S2-82	1172	INASVVNIQKEI	1183	
197	S2-83	1178	NIQKEIDRLNEV	1189	
198	S2-84	1184	DRLNEVAKNLNE	1195	

199	S2-85	1190	AKNLNESLIDLQ	1201	
200	S2-86	1196	SLIDLQELGKYE	1207	
201	S2-87	1202	ELGKYEQYIKWP	1213	
202	S2-88	1208	QYIKWPWYIWLG	1219	
203	S2-89	1214	WYIWLGFIAGLI	1225	N/A
204	S2-90	1220	FIAGLIAIVMVT	1231	N/A
205	S2-91	1226	AIVMVTIMLCCM	1237	N/A
206	S2-92	1232	IMLCCMTSCCSC	1243	N/A
207	S2-93	1238	TSCCSCLKGCCS	1249	
208	S2-94	1244	LKGCCSCGSCCK	1255	N/A
209	S2-95	1250	CGSCCKFDEDDS	1261	
210	S2-96	1256	FDEDDSEPVLKG	1267	
211	S2-97	1262	EPVLKGVKLHYT	1273	
